# A commercial porcine circovirus (PCV) type 2a-based vaccine reduces PCV2d viremia and shedding and prevents PCV2d transmission to naïve pigs under experimental conditions

**DOI:** 10.1016/j.vaccine.2016.11.085

**Published:** 2017-01-05

**Authors:** Tanja Opriessnig, Chao-Ting Xiao, Patrick G. Halbur, Priscilla F. Gerber, Shannon R. Matzinger, Xiang-Jin Meng

**Affiliations:** a The Roslin Institute and The Royal (Dick) School of Veterinary Studies, University of Edinburgh, Midlothian, Scotland, UK; b Department of Veterinary Diagnostic and Production Animal Medicine, College of Veterinary Medicine, Iowa State University, Ames, IA, USA; c College of Biology, Hunan University, Changsha, China; d Department of Biomedical Sciences and Pathobiology, Center for Molecular Medicine and Infectious Diseases, College of Veterinary Medicine, Virginia Polytechnic Institute and State University, Blacksburg, VA, USA

**Keywords:** Porcine circovirus, PCV2, PCV2d, Vaccination, Vaccine efficacy, Transmission

## Abstract

Porcine circovirus type 2 (PCV2) vaccination has been effective in protecting pigs from clinical disease and today is used extensively. Recent studies in vaccinated populations indicate a major PCV2 genotype shift from the predominant PCV2 genotype 2b towards 2d. The aims of this study were to determine the ability of the commercial inactivated PCV2a vaccine Circovac® to protect pigs against experimental challenge with a 2013 PCV2d strain and prevent transmission. Thirty-eight pigs were randomly divided into four groups with 9–10 pigs per group: NEG (sham-vaccinated, sham-challenged), VAC (PCV2a-vaccinated, sham-challenged), VAC + CHAL (PCV2a-vaccinated and PCV2d-challenged), and CHAL (sham-vaccinated, PCV2d-challenged). Vaccination was done at 3 weeks of age using Circovac® according to label instructions. The CHAL and VAC + CHAL groups were challenged with PCV2d at 7 weeks of age and all pigs were necropsied 21 days post-challenge (dpc). The VAC-CHAL pigs seroconverted to PCV2 by 21 days post vaccination (dpv). At PCV2d challenge on 28 dpv, 3/9 VAC and 1/9 VAC + CHAL pigs were seropositive. NEG pigs remained seronegative for the duration of the study. Vaccination significantly reduced PCV2d viremia (VAC + CHAL) at dpc 14 and 21, PCV2d fecal shedding at dpc 14 and 21 and PCV2d nasal shedding at dpc 7, 14 and 21 compared to CHAL pigs. Vaccination significantly reduced mean PCV2 antigen load in lymph nodes in VAC + CHAL pigs compared to CHAL pigs. When pooled serum or feces collected from VAC + CHAL and CHAL pigs at dpc 21 were used to expose single-housed PCV2 naïve pigs, a pooled fecal sample from CHAL pigs contained infectious PCV2 whereas this was not the case for VAC + CHAL pigs suggesting reduction of PCV2d transmission by vaccination. Under the study conditions, the PCV2a-based vaccine was effective in reducing PCV2d viremia, tissue loads, shedding and transmission indicating that PCV2a vaccination should be effective in PCV2d-infected herds.

## 1 Introduction

Porcine circovirus type 2 (PCV2) is a small, non-enveloped, circular-arranged, single-stranded DNA virus that belongs to the *Circoviridae* family [1]. PCV2 is ubiquitous and very resistant to disinfection [2] and most pigs get exposed to PCV2 during their life. In growing pigs, PCV2-infection can be associated with a variety of clinical manifestations commonly summarized as PCV2 associated disease (PCVAD) including systemic illness, enteritis and pneumonia [3]. Porcine dermatitis and nephropathy syndrome (PDNS) has also been linked to PCVAD [4], [5], although definitive experimental proof is still lacking. In addition to PCVAD, PCV2 infection can result in subclinical disease for extended periods of time, which can have a varying impact on pork production [6], [7]. Non-specific clinical signs including reduced weight gain associated with subclinical PCV2 infection are thought to occur due to the effect of PCV2 on the immune system [8].

PCV2 can be classified in five different genotypes including PCV2a, PCV2b, PCV2c, PCV2d and PCV2e of which PCV2a is the oldest [9], [10]. PCV2c has only been identified in archived pig tissues from Denmark [11] and a recent feral pig sample from Brazil [12] and is considered of minor importance. Around 2003 a major genotype shift occurred from PCV2a to PCV2b [11]. Severe PCV2 epidemics linked to PCV2b introduction occurred in North America during 2005/2006 [13] and subsequently led to introduction and large scale usage of PCV2 vaccines in pigs. Today PCV2 vaccination has become a standard management tool in most pig producing areas [14]. Supported by numerous field and experimental trials, PCV2 vaccination has been proven to reduce PCV2 infection, viremia and lesions and increases average daily weight gain (ADG) compared to non-vaccinated pigs [14]. Development of most commercial PCV2 vaccines occurred between 1999 and 2005 when little information on PCV2 genotypes was available and PCV2a was the predominant PCV2 strain at the time. Therefore all major PCV2 vaccines available to date are based on PCV2a [3]. Nevertheless, PCV2a vaccines have been shown to protect pigs against PCV2b challenge in several independent studies [15], [16].

Previously it has been determined that PCV2 has a high mutation rate similar to RNA viruses [17] which may further facilitate rapid emergence and transmission of unique PCV2 genotypes. Furthermore, pigs are often co-infected with multiple PCV2 strains [18], [19]. Since the beginning of this decade a newly recognized genotype, PCV2d, emerged in essentially all large pig populations in North America, South America, Europe and Asia [9], [20]. Moreover, several studies indicate that PCV2d is becoming the predominant strain in the global pig population replacing PCV2a and PCV2b [9], [21]. Frequently, the presence of PCV2d has been linked to PCVAD outbreaks in PCV2-vaccinated herds [22], [23], [24] raising concerns that PCV2 vaccines based on PCV2a strains may not provide sufficient protection against PCV2d strains. The objectives of this study were to determine the ability of a commercial inactivated PCV2a vaccine to protect conventional pigs against experimental challenge and to prevent transmission of a 2013 PCV2d to naïve contact pigs.

## 2 Materials and methods

### 2.1 Ethical statement

The experimental protocol was approved by the Iowa State University Institutional Animal Care and Use Committee (Approval number: 11-14-7900-S).

### 2.2 Animals, housing, and experimental design

**Fig. 1:**
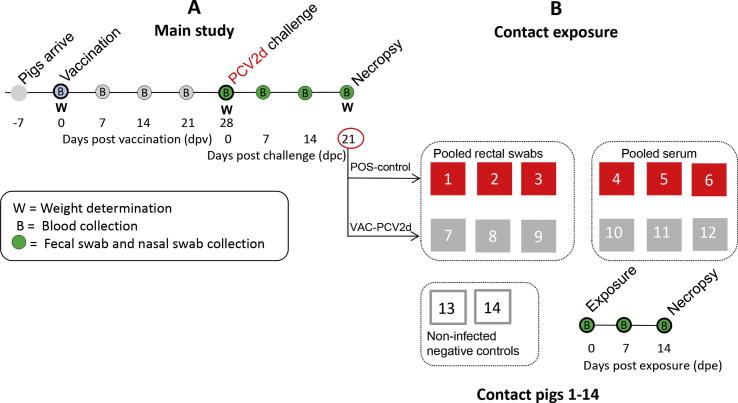
Experimental design and sample collections for the main study (A) and for the contact exposure (B). For the contact exposure, feces and blood collected from the CHAL room or the VAC + CHAL room at dpc 21 were pooled by room and administered by the oral (feces) or intramuscular (serum) route to PCV2 naïve contact pigs which were single housed (one pig per room) in a different building.

Two-week-old, colostrum-fed, crossbred pigs, from a high health commercial breeding herd free of *Mycoplasma hyopneumoniae*, influenza A virus and porcine reproductive and respiratory syndrome virus (PRRSV) and with low PCV2 antibody titers in a portion of the dams and without active PCV2 circulation as evidenced by regular PCV2 PCR testing on pooled serum samples, were purchased for this study. For the main study 38 pigs were randomly assigned to one of four rooms and groups with 9–10 pigs in each group (Table 1). For the contact exposure part of the study, 14 age-matched contact pigs were group-housed in a different room until 7 weeks of age. At that point the contact pigs were moved to individual rooms and were single housed (Fig. 1). Each room contained one pen with one nipple drinker and one self-feeder. All groups were fed *ad libitum* with a balanced, age-appropriate, pelleted feed ration. The experimental design and sample collections are summarized in Fig. 1. Blood was collected in serum separator tubes (BD Vacutainer SST, REF 367088; Fisher Scientific, Pittsburgh, PA, USA), centrifuged at 3000*g* for 10 min at 4 °C, and the serum was stored at −80 °C until testing. Nasal and rectal swabs were collected using polyester swabs and were stored in 5 ml plastic tubes containing 1 ml of sterile saline solution at −80 °C until testing.

### 2.3 Vaccination

At 3 weeks of age (dpv 0 or dpc −28), the VAC and VAC + CHAL pigs were vaccinated intramuscularly in the left neck with 0.5 ml of Circovac® (Merial; Lot No. L404456) as recommended by the manufacturer (Table 1). Similarly, the CHAL and NEG pigs were sham-vaccinated intramuscularly in the left neck with 0.5 ml saline.

### 2.4 Challenge

PCV2d isolate JX535296 [22], [25] was grown to a final titer of 10^4.33^ 50% tissue culture infectious dose (TCID_50_) per ml. At 7 weeks of age (dpv 28 or dpc 0), CHAL and VAC-CHAL pigs (Table 1) received 4.5 ml of the PCV2d challenge virus stock intranasally by slowly dripping 2.25 ml in each nostril. Pigs in the VAC and NEG groups were sham-inoculated with 4.5 ml saline, which was also given intranasally.

### 2.5 Contact pig exposure

Two serum pools were generated by combining serum samples from all VAC-CHAL or all CHAL pigs collected at day post-challenge (dpc) 21. Once combined, 3 ml of the VAC-CHAL dpc 21 serum pool were administered to contact pigs 10, 11, 12 (Fig. 1) by the intramuscular route at day post-exposure (dpe) 0. Similarly, contact pigs 4, 5 and 6 received 3 ml of the CHAL dpc serum pool by the intramuscular route at dpe 0. Fecal material collected on dpc 21 from VAC-CHAL pigs was diluted in phosphate buffered saline (PBS) and contact pigs 7, 8 and 9 each received 8 ml of fecal suspension by the oral route while 8 ml of fecal suspension collected on dpc 21 from CHAL pigs were administered orally to contact pigs 1, 2 and 3. Contact pigs 13 and 14 served as non-infected negative controls (Fig. 1).

### 2.6 Average daily weight gain and clinical observations

All pigs in the main study were weighed at 3 weeks of age (dpv 0 or dpc −28), at 7 weeks of age (dpv 28 or dpc 0) and at 10 weeks of age (dpc 21; Fig. 1). The average daily weight gain was calculated before (dpv 0 to dpv 28) and after PCV2d challenge (dpc 0–21). After PCV2d challenge, all animals were examined daily for signs of illness such as lethargy, respiratory disease, inappetence and lameness.

### 2.7 Serology

Serum samples collected at dpc −28, 0, 7, 14, and 21 for the main study and serum samples collected at dpe 0, 7 and 14 for contact pigs were tested for the presence of anti-PCV2 IgG antibodies by a commercial blocking ELISA (SERELISA® PCV2 Ab Mono Blocking; Zoetis). Samples titers were calculated based on single dilutions using the calculation sheet supplied by the manufacturer.

### 2.8 DNA extraction, detection, quantification and PCV2d confirmation

Total nucleic acids were extracted from all serum samples using the MagMax™ Pathogen RNA Kit (Applied Biosystems, Life Technologies, Carlsbad, CA, USA) on an automated nucleic acid extraction system (Thermo Scientific Kingfisher® Flex, Thermo Fisher Scientific, Pittsburgh, PA, USA) according to the instructions of the manufacturer. All DNA extracts were tested for the presence of PCV2 DNA by a quantitative real-time PCR assay targeting a conserved region in ORF1 as described previously [25], [26]. Samples were considered negative when no signal was observed within the 40 amplification cycles. A differential real-time PCR assay targeting open-reading frame 2 (ORF2) and capable of detecting and differentiating PCV2a, PCV2b and PCV2d was done on all PCV2 PCR-positive pigs at dpc 21 [27]. The differential PCR assay does not react with PCV2c due to a primer mismatch. Selected PCV2 PCR-positive samples were sequenced by using a conventional PCR covering the entire ORF2 as described previously [18] at the Iowa State University DNA Facility, Ames, IA, USA.

### 2.9 Necropsy

At dpc 21 when the pigs in the main study were 10 weeks old, they were euthanized by intravenous pentobarbital sodium overdose (Fatal Plus®, Vortech Pharmaceuticals, LTD, Dearborn, MI, USA) and necropsied. Contact pigs were necropsied at 14 dpe when they were 12 weeks old. As part of a routine necropsy protocol, the extent of macroscopic lung lesions ranging from 0% to 100% was estimated and blindly scored as described previously [28]. The size of superficial inguinal lymph nodes was scored as described previously [29]. Sections of lymph nodes (superficial inguinal, external iliac, mediastinal, tracheobronchial, and mesenteric), tonsil, spleen, kidney, liver, and small intestines (ileum) were collected at necropsy, fixed in 10% neutral-buffered formalin, and routinely processed for histological examination.

### 2.10 Histopathology, immunohistochemistry, and overall lymphoid lesion score

Microscopic lesions were evaluated by a veterinary pathologist blinded to the treatment groups. Lymph nodes, spleen, and tonsil were evaluated for presence and degree of lymphoid depletion and granulomatous replacement of follicles ranging from 0 (normal) to 3 (severe) [30]. Lung sections were scored for the presence and severity of interstitial pneumonia, ranging from 0 (normal) to 6 (severe diffuse) [28]. Sections of ileum, liver and kidney were evaluated for the presence of granulomatous inflammation and scored from 0 (none) to 3 (severe). Immunohistochemistry (IHC) for detection of PCV2 antigen was performed on formalin-fixed and paraffin-embedded sections of lungs, lymph nodes, tonsil, and spleen from all pigs using a rabbit PCV2 polyclonal antiserum [31]. PCV2 antigen scoring was done by a veterinary pathologist blinded to the treatment status. Scores ranged from 0 (no signal) to 3 (more than 50% of lymphoid follicles contained cells with PCV2 antigen staining) [30]. The overall lymphoid lesion score was calculated as described [30]. In brief, a combined scoring system for each lymphoid tissue that ranged from 0 to 9 (lymphoid depletion score 0–3; granulomatous inflammation score 0–3; PCV2 IHC score 0–3) was used.

### 2.11 Statistical analysis

For data analysis, JMP® software version 11.0.0 (SAS Institute, Cary, NC, USA) was used. Summary statistics were calculated for all the groups to assess the overall quality of the data set including normality. Statistical analysis of the data was performed by one-way analysis of variance (ANOVA) for continuous data. A *P*-value of less than 0.05 was set as the statistically significant level. Pairwise test using Tukey’s adjustment was subsequently performed to determine significant group differences. Real-time PCR results (copies per ml of serum) were log_10_ transformed prior to statistical analysis. Statistical analysis for continuous data over time was done by repeated measures multiple analysis of variance (MANOVA). The serology response was assessed between the VAC and VAC + CHAL groups and the PCR results were compared between VAC + CHAL and CHAL groups to determine a time-by-group interaction. Non-repeated nominal data were assessed using a non-parametric Kruskal-Wallis one-way ANOVA, and if significant, pairwise Wilcoxon tests were used to evaluate differences among groups. Differences in incidence were evaluated by using the chi-square test. Percent reduction for the amount of PCV2 DNA was determined as follows: 100 − [(100 × mean log_10_ genomic copies/ml in the vaccinated group) ÷ (mean log_10_ genomic copies/ml in positive control animals)].

## 3 Results

### 3.1 Clinical observation and average daily weight gain (ADG)

No remarkable clinical signs were noted. The ADG is summarized in Table 1. There were no significant differences among groups.

### 3.2 Anti-PCV2 antibody levels

**Fig. 2:**
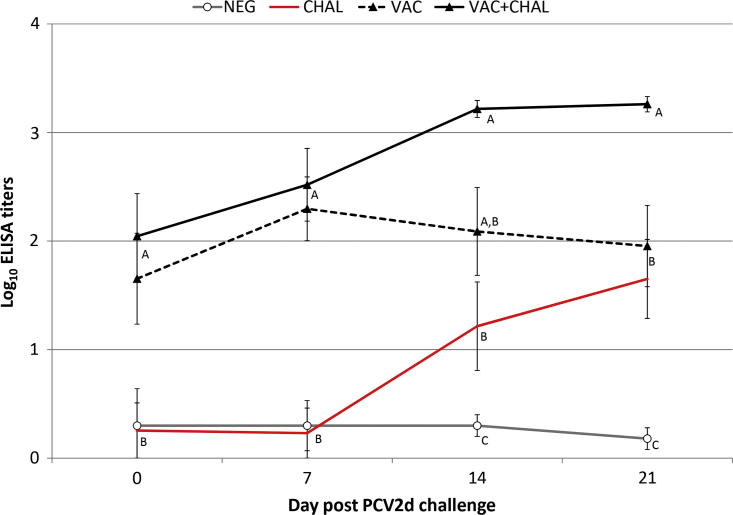
Anti-PCV2 IgG response. Pigs were vaccinated against PCV2 at 3 weeks of age (dpv 0 or dpc −28) and challenged with PCV2d at 7 weeks of age (dpv 28 or dpc 0). Data presented as mean group log_10_ ELISA titer ± SEM. Group means include positive and negative pigs. Significantly different values for a dpc are indicated by different superscripts. The significance level was set to *P* > 0.05.

At dpv 0, dpv 7 and dpv 14 all pigs were negative for PCV2-specific antibodies. One VAC + CHAL pig seroconverted to PCV2 by dpv 21. At dpv 28/dpc 0, PCV2 specific antibodies were detected in 3/9 VAC pigs and 4/9 VAC + CHAL pigs. The group mean log PCV2 ELISA titers from dpc 0 through dpc 21 are summarized in Fig. 2. While the PCV2-antibody titers in VAC + CHAL pigs continued to increase after challenge, titers in non-challenged VAC pigs started to decrease (Fig. 2); the time by group interaction was not significant (F[3, 32] = 0.5914; *P* = 0.38; η2 = 0.64).

### 3.3 Prevalence and amount of PCV2 DNA in serum, rectal swabs and nasal swabs

**Fig. 3:**
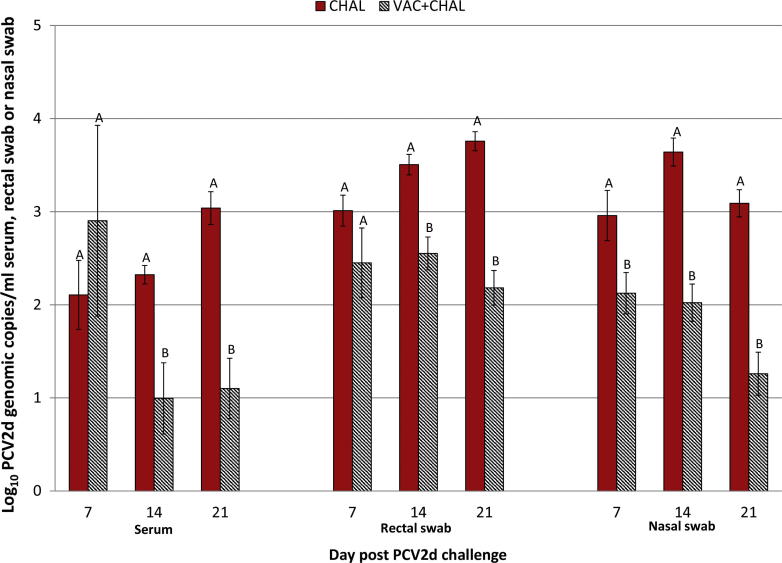
PCV2 viremia, fecal shedding and nasal shedding. Pigs were vaccinated against PCV2 at 3 weeks of age (dpv 0 or dpc −28) and challenged with PCV2d at 7 weeks of age (dpv 28 or dpc 0). Data presented as mean group log_10_ PCV2 genomic copies per ml serum samples, rectal swabs or nasal swabs ± SEM. Group means include positive and negative pigs (considered as 0). Significantly different values for a sample type and dpc are indicated by different superscripts. The significance level was set to *P* > 0.05.

At arrival at the research facility all pigs were negative for PCV2 DNA and all pigs remained PCV2 DNA negative until challenge. There was a significant time by group interaction (F[1, 18] = 4.5882; *P* = 0.04; η2 = 0.53) for PCV2 viremia between the two challenged groups (Fig. 3). After PCV2d challenge, 8/10 CHAL pigs were viremic at dpc 7 and all 10 pigs in this group were viremic at dpc 14 and 21. For the VAC + CHAL pigs, 7/9 were viremic at dpc 7, 6/9 were viremic at dpc 14 and 6/9 were viremic at dpc 21. Group mean genomic copies in serum were significantly (*P* < 0.05) lower for VAC + CHAL pigs compared to CHAL pigs at dpc 14 and 21 (Fig. 3) and by 21 dpc, VAC + CHAL pigs had a 63.8% reduction for the amount of PCV2 DNA in serum compared to the CHAL pigs. In addition, there was a significant time by group interaction (F[1, 22] = 5.4405; *P* = 0.02; η2 = 0.65) for fecal shedding between the two challenged groups. VAC + CHAL pigs had significantly (*P* < 0.05) reduced PCV2d fecal shedding on dpc 14 and dpc 21 compared to CHAL pigs with a 41.9% reduction in PCV2d amount in VAC + CHAL pigs by dpc 21 compared to CHAL pigs. For PCV2 detection in nasal swabs there was a significant time by group interaction (F[2, 28] = 4.3870; *P* = 0.03; η2 = 0.82). By dpc 21 nasal shedding was reduced by 59.2% in VAC + CHAL pigs compared to CHAL pigs (Fig. 3). The PCV2 present in the pigs was confirmed to be PCV2d by PCV2 differential real-time PCR on PCR positive samples collected on dpc 21.

### 3.4 Macroscopic lesions

At necropsy at dpc 21, the lymph nodes appeared mild-to-moderately enlarged in all pigs regardless of treatment status. One VAC + CHAL pig had multiple 0.5–1 cm round-to-oval dark red purple skin lesions in the perineal region, petechial hemorrhage in the cortex area of both kidneys which were tan and slightly enlarged. This pig also had severe chronic gastric ulceration. There were no remarkable macroscopic lesions in any of the other pigs.

### 3.5 Microscopic lesions and PCV2 antigen in tissues

Microscopic lesions are summarized in Table 2. Microscopic lesions in lymphoid tissues were absent in NEG and VAC pigs. In selected PCV2d-infected pigs regardless of vaccination status, there was mild-to-severe lymphoid depletion and histiocytic replacement of follicles. PCV2 antigen in lymph nodes was detected in significantly (*P* < 0.05) more CHAL pigs (6/10) compared to VAC + CHAL pigs (1/9) in lymphoid tissues and the amount of PCV2 antigen was significantly (*P* < 0.05) reduced in vaccinated pigs (Table 2). The distribution of overall lymphoid depletion score category distribution was 3/9 normal, 5/9 mild and 1/9 severe for VAC + CHAL pigs; and 2/10 normal, 5/10 mild, 2/10 moderate and 1/10 severe for CHAL pigs. Individual pigs in all groups regardless of vaccination or infection status had focal-to-multifocal interstitial pneumonia characterized by increased numbers of lymphocytes and macrophages in the alveolar septum and mild type 2 pneumocyte hypertrophy and hyperplasia (score 1). Scores of 2 or 3 were only seen in CHAL and VAC + CHAL pigs. Several PCV2d-infected pigs (1/9 VAC + CHAL pigs and 4/10 CHAL pigs) had PCV2d antigen in the cytoplasm of epithelium cells lining large bronchi and bronchioles in lung tissues (Table 2). The VAC + CHAL pig that had macroscopic lesions consistent with porcine dermatitis and nephropathy syndrome (PDNS) had severe lymphoid depletion and histioctytic replacement of follicles, severe diffuse lymphohistiocytic hepatitis and tubulointerstitial glomerulonephritis with multifocal necrosis of intraglomerular cells and extension of Bowman’s spaces by a homogenous eosinophilic material. PCV2 antigen in this pig was identified in low levels in lymph nodes (score 1) but was not present in renal tissues or liver.

### 3.6 Contact pigs

Contact pigs 1 and 2 exposed to dpc 21 fecal material from the CHAL pigs (Fig. 1) were PCV2 viremic at 14 dpc and also shed PCV2 DNA in nasal and fecal excretions (log_10_ PCV2 genomic copy range from 0.88 to 2.56). PCV2 positive PCR products were sequenced and the presence of the PCV2d strain used to inoculate the pigs in the main study was confirmed (data not shown). PCV2 DNA was not detected in any of the other pigs in samples collected at dpe 0, 7 or 14. None of the contact pigs had seroconverted to PCV2 at study termination.

## 4 Discussion

With the emergence of PCV2d strains in most major swine producing areas across the globe [9], there are concerns over the efficacy of current PCV2a-based commercial vaccines. Previously it has been shown that selected commercial subunit or chimeric PCV2 vaccines can protect pigs against PCV2d challenge [27], [32]. To investigate the ability of Circovac® to protect against PCV2d challenge, pigs were vaccinated at 3 weeks of age and challenged at 7 weeks of age with PCV2d under experimental conditions. This model is representative of what is occurring in the U.S. field where pigs commonly are vaccinated at weaning at 3 weeks of age and get exposed to PCV2 during the nursery period.

The vaccine used in this study, Circovac®, is an inactivated vaccine based on a PCV2a strain isolated in the 1990s and is one of the PCV2 vaccines that has been on the market the longest. Initially this vaccine was developed for usage in adult females in the breeding herd to passively protect suckling and nursery pigs. In 2011, Circovac® was also licensed for use in piglets using the same vaccine preparation but at a lower dose compared to sows (0.5 ml versus 2 ml). In field and experimental studies, Circovac® has been shown to be effective at protecting pigs against the effects of PCV2a and PCV2b challenges when used in sows or piglets [7], [33], [34].

In the present study, at the time of challenge 38.9% of the VAC + CHAL pigs had detectable anti-PCV2 IgG antibody titers which continued to rise after PCV2d challenge and were significantly higher compared to CHAL or VAC pigs at dpc 21. While a detectable antibody response to PCV2 vaccination is a good predictor for successful vaccine administration, it is not necessary to confer protection to pigs. It has been demonstrated that pigs with no detectable humoral response after PCV2 vaccination were protected from subsequent challenge likely due to induction of cellular immunity [35], [36]. While in this study 4/9 VAC + CHAL pigs had detectable antibody titers at challenge, 8/9 VAC + CHAL were protected from subsequent PCV2d challenge and had significantly reduced PCV2d viremia and PCV2d nasal and fecal shedding compared to CHAL pigs indicating that Circovac® confers protection against PCV2d. PCV2d differs from PCV2a by 6.9–9.4% based on amino acids in ORF2. There are several immunodominant regions in ORF2 and PCV2d possess 10 unique amino acid changes in these regions compared to PCV2a strains [9]. Even if one or more of these epitopes would not react with PCV2a antibodies, as Circovac® is based on a full PCV2a strain, antibodies against other regions likely are sufficient to provide protection. In addition, the adjuvant in Circovac®, TS6 Immuneasy™, may have a critical role in protection especially if it can enhance cellular immunity. Cellular immunity was not tested in this study, but in a previous dam vaccination study, Circovac® induced a strong maternally-derived cellular immune response in the offspring of vaccinated sows [37].

One of the VAC + CHAL pigs developed clinical PDNS during this study. To our knowledge this is the first report of PDNS in pigs vaccinated and experimentally infected with PCV2. It has been suggested that excessive PCV2 antibody titers may trigger the development of PDNS [38]. In addition, other etiological agents such as PRRSV and torque teno virus [39] or PCV3 [40] have also been proposed as triggers or causative agents in PDNS. The PDNS-affected pig in this study had no detectable PCV2 antibody titer at challenge and the titer increased towards a low positive level over the following weeks. It also has been suggested that PDNS pigs may have a misdirected, excessive immune response towards a decoy epitope called CP(169–180) which is located in ORF2 of PCV2 [41], [42]. Tests to detect antibodies against CP(169–180) were not available. While PDNS in the past often occurred a few weeks following outbreaks of systemic PCVAD, it appears that PDNS became rare after large-scale introduction of PCV2 vaccination. This suggests that PCV2 vaccination prevents development of PDNS. The reasons why the PCV2 vaccinated pig in this study developed PDNS remain unknown but could include an elevated anti-PCV2 IgM response (which was not tested) or failure to appropriately vaccinate the pig. Alternatively, the gastric ulceration could perhaps have acted as a predisposing factor for a septic event. While PRRSV was not present in the pigs, another un-recognized co-infecting agent could have contributed to the development of PDNS.

PCV2 is ubiquitous, very difficult to remove from a farm and easily transmissible to naïve pigs [2]. In this study, PCV2 in feces collected at dpc 21 was transmissible to naïve pigs from non-vaccinated pigs but not from vaccinated pigs indicating that vaccination reduces PCV2 transmission. This is important considering that routine cleaning procedures on a farm prior to getting a new batch of pigs may not always be sufficient to remove PCV2 [43], [44] and reduction of virus loads by vaccination could assist in preventing transmission of PCV2.

## 5 Conclusions

Under the conditions of this study, PCV2a vaccination reduced PCV2d viremia, PCV2d tissue loads and PCV2d shedding via nasal and fecal routes. In addition, PCV2a-vaccinated and PCV2d-challenged pigs did not transmit PCV2d to naïve contact pigs whereas non-vaccinated PCV2d infected pigs did. PCV2a vaccination was effective against PCV2d challenge.

## Funding

This study was funded by Merial, although the funder had no influence on the experimental design of the study. Additional funding was provided by the Biotechnology and Biological Sciences Research Council (BBSRC) Institute Strategic Programme Grant awarded to the Roslin Institute (BB/J004324/1; BBS/E/D/20241864).

## Conflict of interest statement

The authors declare no financial and personal relationships with other people or organizations that could inappropriately influence this work.

## Figures and Tables

**Table 1:** Experimental groups, treatments at different days post PCV2d challenge (dpc) and average daily weight gain (ADG).

Group designation	Number of pigs	Vaccination	Challenge	ADG^a^	
		*dpc −28*	*dpc 0*	*Vaccination to challenge*	*Challenge to necropsy*
NEG	10	Saline	Saline	463.3 ± 25.2	795.4 ± 40.4
VAC	9	Circovac®	Saline	351.6 ± 29.0	744.7 ± 46.6
VAC + CHAL	9	Circovac®	PCV2d	426.5 ± 21.6	774.0 ± 46.6
CHAL	10	Saline	PCV2d	412.7 ± 26.0	726.0 ± 49.5

a Data presented as group mean ADG in grams ± SEM.

**Table 2:** Group mean microscopic lesions and presence and amount of PCV2 antigen at 21 days post PCV2 challenge.

Group	Lymphoid tissues		Lungs	
	*Overall lymphoid depletion score*^a^	*PCV2 antigen in lymph nodes*^b^	*Interstitial pneumonia*^c^	*PCV2 antigen in lung tissues*^b^
NEG	0.1 ± 0.1^A^^,^^d^	0/10 (0.0 ± 0.0)^A^	0.4 ± 0.2^A^	0/10 (0.0 ± 0.0)
VAC	0.3 ± 0.2^A,B^	0/9 (0.0 ± 0.0)^A^	0.6 ± 0.2^A,B^	0/9 (0.0 ± 0.0)
VAC + CHAL	1.6 ± 0.7^B^	1/9 (0.1 ± 0.1)^A^	0.7 ± 0.3^A,B^	1/9 (0.1 ± 0.1)
CHAL	2.5 ± 0.7^B^	6/10 (0.9 ± 0.3)^B^	1.4 ± 0.3^B^	4/10 (0.1 ± 0.1)

a Overall lymphoid depletion score ranging from 0 = normal to 9 = severe.

b Determined by immunohistochemistry ranging from 0 = absent to 3 = abundant antigen.

c Interstitial pneumonia score ranging from 0 = normal to 6 = severe.

d Data presented as group mean ± SEM or as prevalence (group mean ± SEM). Groups connected by different superscripts within a column have significantly (*P* < 0.05) different group means.
